# Comprehension of Top 200 Prescribed Drugs in the US as a Resource for Pharmacy Teaching, Training and Practice

**DOI:** 10.3390/pharmacy6020043

**Published:** 2018-05-14

**Authors:** Andrea V. Fuentes, Moises D. Pineda, Kalyan C. Nagulapalli Venkata

**Affiliations:** Department of Pharmaceutical Sciences, College of Pharmacy, Larkin University, Miami, FL 33169, USA; afuentes@myularkin.org (A.V.F.); mpineda@myularkin.org (M.D.P.)

**Keywords:** black box warning, oral administration, cardiovascular, central nervous system, endocrine, gastrointestinal, antibiotics, antihypertensive, inhalers, biologics, nasal, injection

## Abstract

Pharmacists have access to a plethora of information related to drugs. Online compendia concerning top 200 prescribed drugs are readily-accessible, comparatively-easy to search. While these resources provide some information about the commonly prescribed drugs, they lack in furnishing in-depth knowledge to pharmacy students, pharmacists and other healthcare professionals. The aim of this paper is to present the relevant details of top 200 most prescribed drugs in the United States. The names and therapeutic classes of top 200 prescribed drugs were compiled from online resources. The pharmacological actions of drugs, any reported adverse reactions and black box warnings are collected from drug bank resources, such as AccessPharmacy and Lexicomp. The paper provides comprehensive information about top 200 prescribed drugs, which includes generic names, pharmacological action, route of administration and adverse reaction profile including black box warning when applicable. Overall, the drug list may serve as an easy access of ideas for pharmacists, researchers and other healthcare professionals interested in developing new strategies for treating patients with various ailments.

## 1. Introduction

Over the past few decades, the health care needs of our population changed along with the role of pharmacists [1]. Historically, pharmacists’ role in healthcare was centered on dispensing medications and ensuring the accurate delivery of medications to patients. In addition to allocating medications and safeguarding patient safety, today pharmacists are an integral part of our health care team and also are considered the most accessible health care professionals [2]. This approachability enables them to perform their pharmacists’ patient care process (PPCP), such as collect, access, plan, implement and follow-up to monitor and evaluate the appropriateness and effectiveness of medications and obtain patient feedback [3]. Additionally, pharmacists advise other health professionals concerning medication therapy decisions, the composition of drugs, their physicochemical and biological properties. Pharmacists also ensure the drug purity, efficacy, their interactions and side effects [4].

As per the survey conducted by National Pharmacist Workforce in 2014, over a decade pharmacists providing medication therapy management increased from 13% to 60% and those performing immunizations incremented from 15% to 53% respectively [5,6].

To deliver excellent pharmacy services to patients, pharmacists need to have complete knowledge of commonly prescribed drugs [7,8]. In 2014, the total number of prescriptions dispensed were approximately 4.325 billion, out of which the top 200 most prescribed drugs accounted for approximately 2.87 billion [9,10]. The top 200 drugs represent 66.6% (2/3) of total prescriptions filled in the US. The topic on top 200 most prescribed drugs in the US has been previously compiled in number of resources [11,12]. They offer a short comprehensive review of this topic [13,14]. However, in order to maximize studying, these guide/books/chapters, it is critical that a student has a firm grasp on the complete knowledge of the most commonly used medications [15]. This includes generic drugs as wells as mechanism of action (MOA), side effects, first line therapy indication, black box warning, and most common routes of administration. Therefore, the purpose of this article is to summarize the most commonly prescribed medications in the US and provide pharmacists and pharmacy students a resource before undertaking the task of practicing and studying for North American Pharmacist Licensure Examination (NAPLEX).

## 2. Materials and Methods 

**Figure 1:**
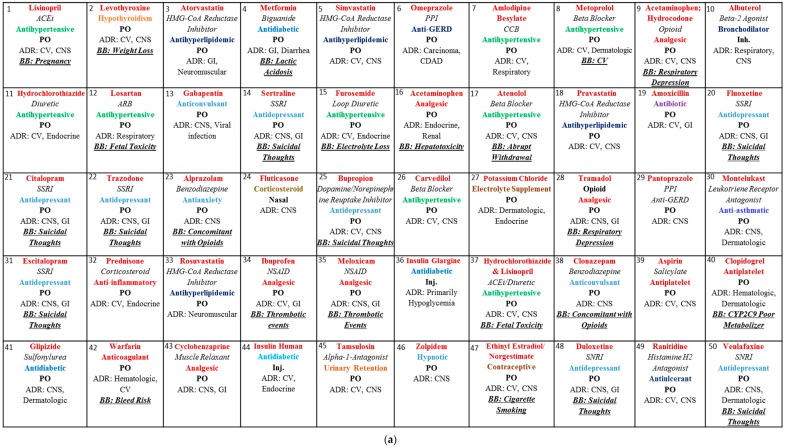

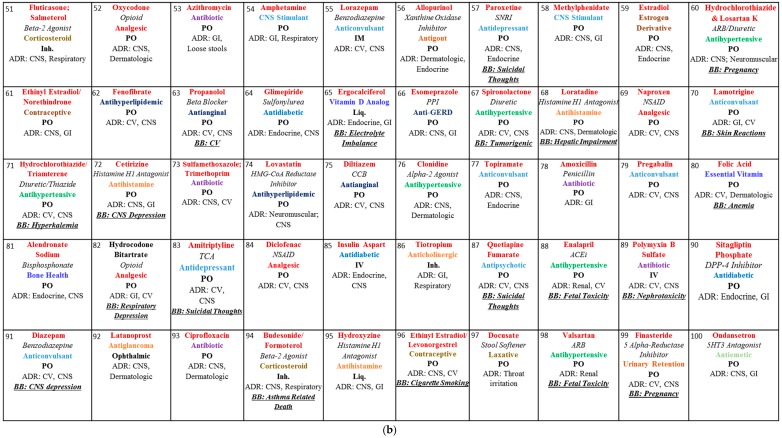

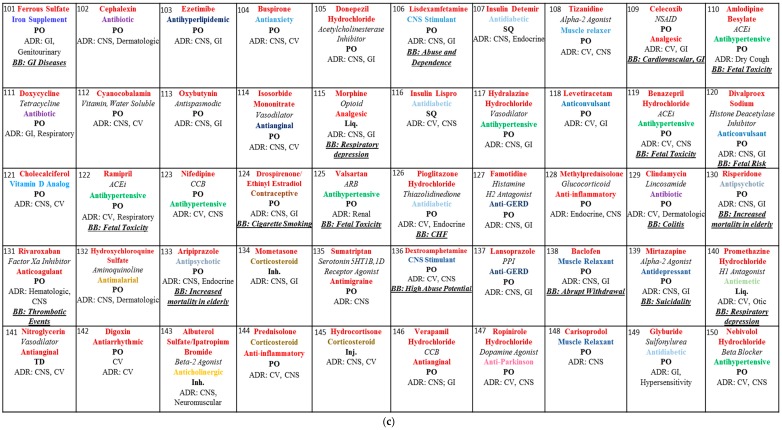

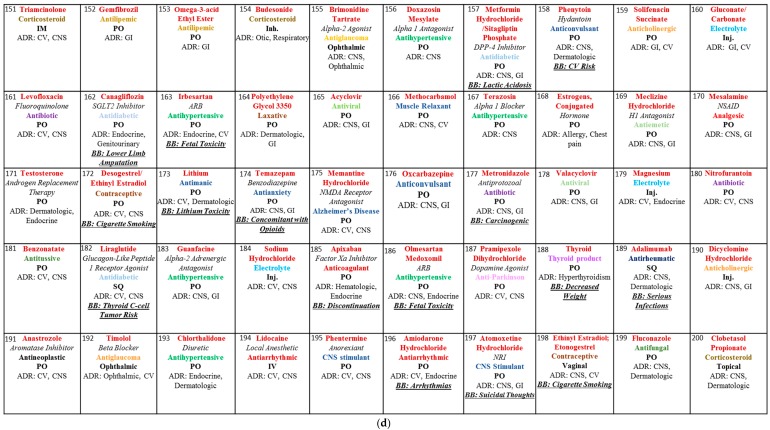
(**a**) List of 1–50 most prescribed drugs; (**b**) List of 51–100 most prescribed drugs; (**c**) List of 101–150 most prescribed drugs; (**d**) List of 151–200 most prescribed drugs.

To accomplish the study objectives, this study was divided into two phases. Phase I consisted of gathering information on the drug names and therapeutic classes, which were compiled from the Clincalc.com. The Clinicalc.com website obtains its data annually from medical expenditure panel survey [MEPS] which is conducted by the US government [10]. Phase II entails collecting information on the drugs, their pharmacological actions, adverse reactions, and any possible black box warnings from resources, such as Clinical Drug Information from AccessPharmacy database on drug monographs and Lexicomp [16,17]. The prescribed drugs in the Figure 1a–d are numerically arranged based on the number of prescriptions filled and dispensed for each generic drug in the US. A set of inclusion and exclusion criteria was developed to select 200 commonly prescribed drugs. We included generic drugs obtained from the ClinCalc website, pharmacological actions and drug classes when applicable, most frequently used routes of administration, top two body systems affected by adverse drugs reactions, and the most advocated black box warming. Chemicals and biologics are included. The drugs not listed as top 200 drugs in the ClinCalc website were excluded. Additionally, if a drug is used in combination with another drug it is treated as a separate drug entity from the parent drug. 

## 3. Results and Discussion

**Figure 2:**
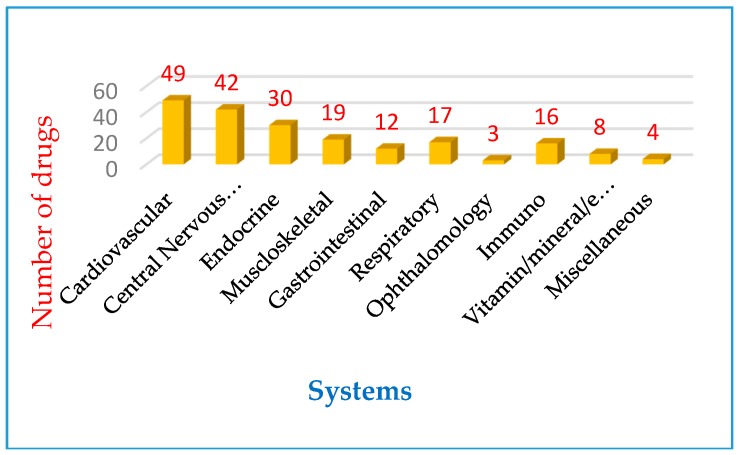
Lists the number drugs prescribed for each system.

Top 200 most prescribed drugs shown in Figure 1a–d, were developed using the data obtained from Clincalc website. The individual drugs are represented by generic name, drug class (when applicable), pharmacological action, major route of administration, adverse drug reactions and any applicable black box warnings [BB]. The lists contain many blockbuster drugs of the last 10 to 15 years, such as atorvastatin, simvastatin, etc. The most prescribed drugs based on systems were cardiovascular (49), central nervous system (42), endocrine (30) and musculoskeletal (19). They accounted for approximately 140 drugs (70%) of top 200 most prescribed drugs. Drug utilization by systems is shown in Figure 2.

### 3.1. Blackbox Warning

As per FDA regulations any drug that may lead to adverse reactions and that might cause serious injury or result in death should be labeled by black box warning [18]. The number of drugs with black box warning are 81 drugs (40.5%) of 200 most prescribed medicines. 

### 3.2. Dosage Forms

The lists also highlights the dosage forms of top 200 most prescribed drugs, they were oral, PO (166), inhalation, inh (7), intravenous, IV (3), intramuscular, IM (2), injections, inj (7), liquids, liq (4), subcutaneous, SQ (4), ophthamological, ophth (3), nasal, NAS (1), topical, TOP (1), transdermal, TM (1) and vaginal, VAG (1). 

### 3.3. Biologicals and Chemicals

As per the lists, only 7 (3.5%) of drugs were biologicals among the top 200 most prescribed drugs, rest were chemical entities.

### 3.4. Opioids

Five opioids namely, acetaminophen/hydrocodone, tramadol, oxycodone, hydrocodone, and morphine are among the top 200 most prescribed drugs. In fact, Aacetaminophen/hydrocodone is 1 of the top 10 most prescribed drugs. Tramadol and oxycodone are listed among top 60 most prescribed drugs in the US.

### 3.5. Adverse Drug Reactions

**Figure 3:**
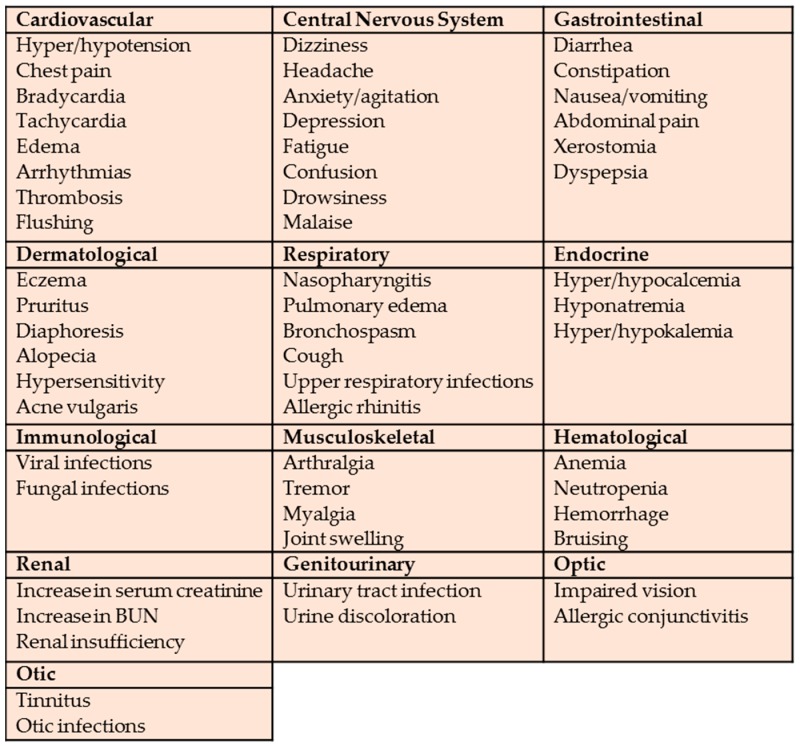
The most common adverse drug reactions for top 200 drugs by systems

Within top 200 drugs the most common ADRs and their range according to the systems are shown in Figure 3. 

## 4. Conclusions

The visual language of the top 200 most prescribed drugs presented in the paper will foster long-term learning and enable students and residents to be more confident and competent before facing actual patients. Also will provide a quick reference about their therapeutic use, side effects, dosage forms and black box warning information of 66% of the total drugs prescribed in the US. Additionally, the drug lists will be handy for pharmacists, researchers and other healthcare professionals interested in developing new strategies for treating patients with various ailments.

## Abbreviations

CV	Cardiovascular
GI	Gastrointestinal
IM	intramuscular
Opth	Ophthalmic
Inj	Injection
ADR	Adverse Drug Reaction
CNS	Central Nervous System
PO	Oral
IV	Intravenous
Inh	Inhalation
Liq	Liquid
BB	Black Box Warning
SQ	Subcutaneous
TD	Transdermal
CDAD	Clostridium Difficile Associated Diarrhea
GERD	Gastroesophageal Reflux Disease
ACEi	Angiotensin-Converting-Enzyme Inhibitor
ARB	Angiotensin II Receptor Blockers
HMG-CoA Reductase Inhibitor	3-hydroxy-3-methyl-glutaryl-coenzyme A reductase Inhibitor
PPI	Proton Pump Inhibitor
CCB	Calcium Channel Blocker
SSRI	Selective Serotonin Reuptake Inhibitors
SNRI	Serotonin–Norepinephrine Reuptake Inhibitors
TCA	Tricyclic Antidepressant
NRI	Norepinephrine Reuptake Inhibitor
NSAID	Non-Steroidal Anti-Inflammatory Drug
SGLT2 Inhibitor	Sodium-Glucose co-Transporter-2 Inhibitor
DPP-4 Inhibitor	Dipeptidyl Peptidase-4 Inhibitor
